# Editorial: Inhibitory Receptors and Pathways of Lymphocytes

**DOI:** 10.3389/fimmu.2020.01552

**Published:** 2020-07-22

**Authors:** Alexandre M. Carmo, Paul E. Love, Aaron J. Marshall

**Affiliations:** ^1^IBMC - Instituto de Biologia Molecular e Celular, Porto, Portugal; ^2^i3S - Instituto de Investigação e Inovação em Saúde, Universidade do Porto, Porto, Portugal; ^3^Section on Hematopoiesis and Lymphocyte Biology, Eunice Kennedy Shriver National Institute of Child Health and Human Development, National Institutes of Health, Bethesda, MD, United States; ^4^Department of Immunology, Rady Faculty of Health Sciences, University of Manitoba, Winnipeg, MB, Canada

**Keywords:** lymphocyte, inhibitory receptor, inhibitory signaling, checkpoint blockade, immunotherapy, autoimmunity, cancer

Antigen receptor recognition is a key event that both initiates lymphocyte activation and impacts the fate of immune responses. Co-receptors and co-stimulatory molecules coordinate with antigen receptors to amplify and transduce the initial signals initially triggered at the cell surface, and integrate subsequent signals required to sustain responses. Equally important are the molecular mechanisms that control and terminate activation responses, which act at multiple levels to attenuate activation, modulate signaling thresholds for activation, and shutdown responses in the face of chronic antigen receptor engagement. A very fine line separates activation-mediated clonal expansion from cell death by apoptosis at all stages of lymphocyte development and inhibitory receptors and their associated signaling molecules can determine the difference between life and death. Lymphocyte differentiation and acquisition of effector functions is continuously modulated by a variety of receptors and signaling pathways which balance quiescent, activated and exhausted phenotypes as well as cytolytic activity and cytokine production patterns. Inhibitory receptors and signaling molecules collectively have critical functions to integrate multiple environmental inputs to modulate immune cell activation states in a variety of tissues. As discussed in the reviews in this Topic, inhibitory receptor function can tip the balance between health and disease in a variety of contexts involving chronic lymphocyte activation.

Several reviews in this Topic address the biological functions and clinical applications of Ig superfamily “checkpoint receptors” PD-1, CTLA-4, BTLA, TIGIT, 2B4, Tim3, and LAG-3. De Sousa Linhares et al. provide an excellent overview, summarizing current evidence regarding the unique features and clinical significance of many of these receptors, as well as discussing the remaining open questions. Yasuma-Mitobe and Matsuoka discuss studies implicating these receptors in retroviral infections such as HIV. Morris et al. discuss the function of these inhibitory receptors in the context of CD8+ T cell memory, recall responses and terminal differentiation. Brunner-Weinzierl and Rudd discuss the roles of PD-1 and CTLA-4 in tumor immunotherapy, focussing on their roles in controlling T cell migration via impact on integrin activation and chemokine receptor expression and signaling. Gianchecchi and Fierabracci comprehensively review evidence regarding the roles of PD-1 and its ligand PD-L1 in regulatory T cell (Treg) development and functional activity, and address how this may relate to development of autoimmunity. Paluch et al. address the role of both PD-1 and CTLA-4 in autoimmunity, discuss the rationale for checkpoint receptor agonists as treatment for this class of diseases and outline several different approaches being used in the design of checkpoint agonists.

The article by Georgiev et al. provides a focused review of CD96, another type 1 transmembrane glycoprotein of the Ig superfamily. CD96, together with TIGIT and other receptors, form a distinct sub-group of regulatory Ig superfamily receptors that are relatively less studied, but likely play regulatory roles in T cells and NK cells. Kim and Kim also address the role of CD96 in NK cell function and discuss the role of established checkpoint receptors such as PD-1, CTLA-4, TIM-3, and LAG-3 in NK cells. These authors also address the role of killer inhibitory receptors (KIR) and CD94/NKG2A in mediating the unique regulatory mechanisms of NK cells.

Gonçalves et al. and Voisinne et al. address the scavenger receptor cysteine-rich (SRCR) glycoproteins CD5 and CD6 and their differential roles in T cell development and responses. The significance of ligand binding in signal modulation is explored, as well as the inhibitory signaling mechanisms. The therapeutic potential of targeting these receptors for immunotherapy is discussed.

Sialic acid-binding Ig-like lectins (Siglecs) are another important family of inhibitory receptors in lymphocytes. Tsubata has reviewed data regarding the roles of the Siglec receptors CD22, CD72, and SiglecG in development and function of B cells, focusing on how distinct ligand-recognition properties of these receptors determine their functional roles. Clark and Giltiay comprehensively review literature regarding the roles of CD22 in B cell activation, migration, tolerance and autoimmunity, as well as evidence regarding therapeutic targeting of CD22.

Different classes of signaling molecules mediate the inhibitory functions of these receptors. Pike and Tremblay review the roles of protein tyrosine phosphatases such as PTPN22 in controlling CD4+ T cell activation and function, particularly in the context of intestinal inflammation and inflammatory bowel disease. The role of SHP-1 protein tyrosine phosphatase recruitment via receptor ITIM motifs is touched on by several other reviews, while some authors indicate that early implication of this phosphatase turned out to be incorrect (for example, in the case of CD5). The role of cbl family ubiquitin ligases in CD5/6 function is explored by Voisinne et al. and Gonçalves et al.. These authors also touch on the roles of the inhibitory tyrosine kinase Csk which controls the activity of Src kinases.

Rodríguez-Galán et al. uniquely explore the interesting topic of immune regulation mediated by miRNA networks. They discuss evidence that miRNAs can control not only expression of inhibitory receptors such as PD-1 and CTLA-4 in lymphocytes, but also inhibitory protein phosphatases and phosphoinositide phosphatases, critical cell survival and cell cycle regulators as well as co-stimulatory receptors and cytokines.

Together, the excellent reviews collected under this Topic only begin to capture some of the breadth and depth of research in this fascinating and important area. It is clear that “Inhibitory receptors” is somewhat of an over-simplification to describe the nuances of how these receptors function to maintain homeostasis within the immune system. Their roles extend beyond modulation of initial lymphocyte activation signals, with perhaps their greatest impact being in balancing the needs of host defense, avoiding excessive immunopathology, and maintaining normal physiology during infection and chronic inflammation. Future studies will no doubt address how these receptors each function within different tissue contexts and in different contexts of physiological and metabolic stress. This will require better understanding of individual receptor/ligand dynamics and how expression of receptors and ligands are modulated in different locations and contexts.

Therapeutic targeting of inhibitory circuits in lymphocytes will no doubt continue to be a major focus over the coming years. Creative approaches to targeting at the level of receptors, ligands and signaling molecules will be guided by improved understanding of the relevant molecular and cell biology. One issue in therapeutic targeting is the clear functional redundancy among some receptors and ligand systems, which may require targeting multiple molecules to achieve biological impact. Targeting at the level of inhibitory signaling molecules may in some cases offer opportunities to bypass receptors and manipulate lymphocyte functions via small molecule inhibitors or activators of signaling molecules, or perhaps miRNAs. A limitation that has become very apparent with therapeutic targeting of checkpoint receptors is that disturbing the immune ecosystem by blocking inhibitory pathways can have unintended consequences. Freeing the immune system from control networks that have evolved to maintain homeostasis may cure one disease, but create another. This underlines the need for a deeper understanding of how these inhibitory networks function at the molecular, cellular, tissue and whole organism level. Ultimately the goal is to more selectively release or restore these powerful regulatory systems to reset the ecosystem for maximum therapeutic benefit.

## Author Contributions

AC, PL, and AM served as co-editors of this topic and wrote the editorial. All authors contributed to the article and approved the submitted version.

## Conflict of Interest

The authors declare that the research was conducted in the absence of any commercial or financial relationships that could be construed as a potential conflict of interest. The handling editor declared a shared affiliation, though no other collaboration, with one of the authors PL at time of review.

